# Supplementary material for: An open-source framework for large-scale, flexible evaluation of biomedical text mining systems
Source: J Biomed Discov Collab. 2008 Jan 29;3:1. doi: 10.1186/1747-5333-3-1 (PMC2276192; doi:10.1186/1747-5333-3-1)
Supplement: Additional file 1 — Evaluation platform source code. The additional file contains the software evaluation framework discussed in this paper. The most current release can be downloaded from SourceForge.net [1]. See the accompanying README file for instructions on installation and use. Also included in the distribution is a collection of UIMA wrappers for some commonly used BioNLP tools and annotated corpora. [file 1747-5333-3-1-S1.zip › bionlp_uima_repository-1.0.1/docs/api/edu/uchsc/ccp/uima/ae/nlp/ei/gene/ABNER_AE.html]

ABNER\_AE (Center for Computational Pharmacology Util/UIMA API)


|  |  |  |
| --- | --- | --- |
| **Overview** \| **Package** \| **Class** \| **Tree** \| **Deprecated** \| **Index** \| **Help** | |  |
| **PREV CLASS**   **NEXT CLASS** | **FRAMES**    **NO FRAMES**     **All Classes** |  |
| SUMMARY: NESTED \| FIELD \| CONSTR \| METHOD | DETAIL: FIELD \| CONSTR \| METHOD |  |


---


## edu.uchsc.ccp.uima.ae.nlp.ei.gene Class ABNER\_AE

```
java.lang.Object
  org.apache.uima.analysis_engine.annotator.Annotator_ImplBase
      org.apache.uima.analysis_engine.annotator.JTextAnnotator_ImplBase
          edu.uchsc.ccp.uima.ae.nlp.ei.gene.ABNER_AE
```

**All Implemented Interfaces:**: org.apache.uima.analysis\_engine.annotator.BaseAnnotator, org.apache.uima.analysis\_engine.annotator.JTextAnnotator

---

``` public class ABNER_AE extends org.apache.uima.analysis_engine.annotator.JTextAnnotator_ImplBase ```

The class incorporates the ABNER entity identification system into the UIMA analysis engine framework. This class outputs 5 distinct
annotation types. In the CCPTypeSytem, all five types fall under the SemanticAnnotation classification.

**Author:**
:   William A. Baumgartner, Jr.

---

| **Field Summary** | |
| --- | --- |
| `static String` | `PARAM_USE_BIOCREATIVE_MODEL` |


**Constructor Summary**

`ABNER_AE()`


| **Method Summary** | |
| --- | --- |
| `void` | `initialize(org.apache.uima.analysis_engine.annotator.AnnotatorContext ac)` |
| `void` | `process(org.apache.uima.jcas.JCas jcas, org.apache.uima.analysis_engine.ResultSpecification arg1)`             This method employs the ABNER entity identification system to search the document text for Gene, RNA, DNA, Cell Line, and Cell Type mentions. |

**Methods inherited from class org.apache.uima.analysis\_engine.annotator.Annotator\_ImplBase**

`destroy, finalize, getContext, getTypeSystem, reconfigure, typeSystemInit`

**Methods inherited from class java.lang.Object**

`clone, equals, getClass, hashCode, notify, notifyAll, toString, wait, wait, wait`

**Methods inherited from interface org.apache.uima.analysis\_engine.annotator.BaseAnnotator**

`destroy, reconfigure, typeSystemInit`

**Field Detail**

### PARAM\_USE\_BIOCREATIVE\_MODEL

```
public static final String PARAM_USE_BIOCREATIVE_MODEL
```

**See Also:**: Constant Field Values


**Constructor Detail**

### ABNER\_AE

```
public ABNER_AE()
```


**Method Detail**

### initialize

```
public void initialize(org.apache.uima.analysis_engine.annotator.AnnotatorContext ac)
                throws org.apache.uima.analysis_engine.annotator.AnnotatorInitializationException,
                       org.apache.uima.analysis_engine.annotator.AnnotatorConfigurationException
```

:   **Specified by:**: `initialize` in interface `org.apache.uima.analysis_engine.annotator.BaseAnnotator` **Overrides:**: `initialize` in class `org.apache.uima.analysis_engine.annotator.Annotator_ImplBase`
:   **Throws:**: `org.apache.uima.analysis_engine.annotator.AnnotatorInitializationException`: `org.apache.uima.analysis_engine.annotator.AnnotatorConfigurationException`

---


### process

```
public void process(org.apache.uima.jcas.JCas jcas,
                    org.apache.uima.analysis_engine.ResultSpecification arg1)
             throws org.apache.uima.analysis_engine.annotator.AnnotatorProcessException
```

:   This method employs the ABNER entity identification system to search the document text for Gene, RNA, DNA, Cell Line, and Cell Type
    mentions.

    :   **Throws:**: `org.apache.uima.analysis_engine.annotator.AnnotatorProcessException`


---


|  |  |  |
| --- | --- | --- |
| **Overview** \| **Package** \| **Class** \| **Tree** \| **Deprecated** \| **Index** \| **Help** | |  |
| **PREV CLASS**   **NEXT CLASS** | **FRAMES**    **NO FRAMES**     **All Classes** |  |
| SUMMARY: NESTED \| FIELD \| CONSTR \| METHOD | DETAIL: FIELD \| CONSTR \| METHOD |  |


---
